# Enhancing the Mechanical Properties of Co-Cr Dental Alloys Fabricated by Laser Powder Bed Fusion: Evaluation of Quenching and Annealing as Heat Treatment Methods

**DOI:** 10.3390/ma17215313

**Published:** 2024-10-31

**Authors:** Bartlomiej Konieczny, Agata Szczesio-Wlodarczyk, Artur Andrearczyk, Bartlomiej Januszewicz, Sebastian Lipa, Rafał Zieliński, Jerzy Sokolowski

**Affiliations:** 1 University Laboratory of Materials Research, Medical University of Lodz, Pomorska 251 St., 92-213 Lodz, Poland; bartlomiej.konieczny@umed.lodz.pl; 2 Department of Turbine Dynamics and Diagnostics, Institute of Fluid-Flow Machinery, Polish Academy of Sciences, Fiszera 14 St., 80-231 Gdansk, Poland; 3 Institute of Material Science and Engineering, Lodz University of Technology, 1/15 Stefanowskiego St., 90-537 Lodz, Poland; 4 Stomatologia na Księżym Młynie, 16D Tymienieckiego St., 90-365 Lodz, Poland; 5 Department of General Dentistry, Medical University of Lodz, Pomorska 251 St., 92-213 Lodz, Poland

**Keywords:** SLM, Co-Cr, dental, postprocessing, isothermal, athermal

## Abstract

Residual stresses and anisotropic structures characterize laser powder bed fusion (L-PBF) products due to rapid thermal changes during fabrication, potentially leading to microcracking and lower strength. Post-heat treatments are crucial for enhancing mechanical properties. Numerous dental technology laboratories worldwide are adopting the new technologies but must invest considerable time and resources to refine them for specific requirements. Our research can assist researchers in identifying thermal processes that enhance the mechanical properties of dental Co-Cr alloys. In this study, high cooling rates (quenching) and annealing after quenching were evaluated for L-PBF Co-Cr dental alloys. Cast samples (standard manufacturing method) were tested as a second reference material. Tensile strength, Vickers hardness, microstructure characterization, and phase identification were performed. Significant differences were found among the L-PBF groups and the cast samples. The lowest tensile strength (707 MPa) and hardness (345 HV) were observed for cast Starbond COS. The highest mechanical properties (1389 MPa, 535 HV) were observed for the samples subjected to the water quenching and reheating methods. XRD analysis revealed that the face-centered cubic (FCC) and hexagonal close-packed (HCP) phases are influenced by the composition and heat treatment. Annealing after quenching improved the microstructure homogeneity and increased the HCP content. L-PBF techniques yielded superior mechanical properties compared to traditional casting methods, offering efficiency and precision. Future research should focus on fatigue properties.

## 1. Introduction

New technologies for processing metallic materials such as cobalt-chromium alloys are becoming increasingly popular among dentists and dental technicians. Computer-aided design and manufacturing (CAD–CAM) is continually replacing traditional methods such as lost wax casting [1,2]. These new technologies allow the creation of three-dimensional (3D) structures using patient data obtained by special imaging devices (e.g., optical scanners) [3,4]. In CAD–CAM, two types of processing can be distinguished: subtractive manufacturing (e.g., milling) and additive manufacturing, known as 3D printing (e.g., laser powder bed fusion, fused filament deposition, light curing technology) [5,6,7]. Thanks to 3D printing technology, new possibilities in dentistry are available, such as the production of complex customized products, especially when metallic materials are needed [8].

Laser powder bed fusion (L-PBF) uses a high-quality laser to melt metallic powder layer by layer to form a 3D object, according to a digital project [9]. Several factors determine the final properties of the L-PBF product. The build orientation and the selection of the machine parameters (laser beam size and power, scanning speed, layer thickness) are very important aspects [10,11,12,13,14]. However, it is worth emphasizing that this method is not without drawbacks. The thermal gradients during manufacturing result in high internal stresses in L-PBF products [15,16]. Furthermore, the as-built products are characterized by anisotropic columnar grains and sub-grains with dendritic structures. Due to these reasons, an additional heat treatment is required to reduce the internal stress and ensure a uniform microstructure [17,18,19,20,21].

Dental products made using L-PBF technology are often subjected to heating and slow cooling in an oven. The homogenization of the material microstructure increases with increasing thermal treatment temperature (from 750 °C to 1050 °C). However, even prolonged six hours of postprocessing up to 1050 °C is insufficient for eliminating anisotropy and residual stress. To obtain total homogenization of the sample’s microstructure and eliminate residual stress, L-PBF-processed Co–Cr alloys should be heated to at least 1150 °C [22,23].

Various thermal treatment methods are employed to optimize the performance of L-PBF products. These methods not only affect the internal structure of the materials but also significantly influence the formation of crystallographic phases. Co matrix in Co-Cr alloys may consist of both the high-temperature face-centered cubic (fcc) phase and the low-temperature stable hexagonal close-packed (hcp) phase. The FCC phase provides greater ductility, and the HCP phase offers higher strength and hardness. Therefore, the ratio between FCC and HCP will have a significant impact on the material properties [24,25]. In Co-Cr alloys, the formation of the hexagonal close-packed (HCP) phase can occur through two main mechanisms: athermal (by quenching) and isothermal (by annealing process) transformation [26].

Quenching is a standard thermal treatment of metals that has the potential to induce significant changes in the microstructure, which can alter the material’s mechanical properties [27]. Annealing after quenching may further modify the material properties, presenting additional opportunities for improving the alloy’s characteristics. Temperatures used in isothermal aging range from 650 to 950 °C [28]. For Co-Cr alloys, especially those used in dentistry, there are only a few studies on the impact of this thermal treatment method [29,30]. Such research is essential for developing optimized heat treatment protocols that enhance the performance of L-PBF-fabricated dental components, ensuring they meet the high standards required for dental components.

In the present work, samples were produced by L-PBF using a 45° build orientation to the build direction. In addition, cast samples (standard manufacturing method) were tested as a second reference material. This study aimed to evaluate the influence of selected heat treatments on the resulting microstructure, tensile strength, and hardness of L-PBF-processed Co–Cr alloys. In addition to the homogenization of the microstructure and stress relief of the samples, the authors hope to improve the mechanical properties. The following null hypotheses were evaluated: (1) there are no differences in the structure of Co-Cr alloys or their mechanical properties after quenching or quenching with annealing; (2) the properties of Co-Cr samples prepared with the lost wax casting method are comparable to those of L-PBF samples.

## 2. Materials and Methods

### 2.1. The Sample Preparation

Commercially available Co–Cr–Mo alloy powder, Starbond COS (Scheftner, Mainz, Germany), and two alloy ingots, Starbond COS and MoguCera (Scheftner, Mainz, Germany), were used in this study. The manufacturers’ information about the chemical composition of the materials used is shown in Table 1. Two types of samples were made. First, dumbbell-shaped samples (3 mm in diameter and 18 mm in gage length, *n* = 7/group) were used, and second, cuboid samples with dimensions (4.5 mm height, 10 mm width, and 10 mm length, *n* = 3/per group) were used.

Casting Co-Cr samples were manufactured by the traditional lost wax technique. Two Co-Cr samples were produced using the lost wax casting method. To ensure stability and dimensional repeatability, the samples were designed in CAD software (Fusion 360, ver. 2.0.20478 Autodesk, San Rafael, CA, USA) based on a standard and then cut on a milling machine (CORiTEC 350 loader PRO PLUS, Imes-Icore GmbH, Hessen, Germany) from a wax disc. The mold was prepared by securing the milled wax sample patterns in a silicone ring using wax channels with a diameter of 3 mm and an electric knife. A surface tension-reducing agent at the wax-casting interface was used to eliminate the formation of air bubbles on the wax surface and prevent casting distortions. The wax structure placed in the silicone casting ring was submerged in a covering phosphate investment material (Bellavest SH, BEGO Bremer Goldschlägerei Wilh. Herbst GmbH & Co. KG, Bremen, Germany) according to the manufacturer’s instructions. To melt/remove the wax, the prepared casting mold was placed in a burnout furnace (Magma, Renfert GmbH Company, Hilzingen, Germany) heated to a temperature of 900 °C. Then, the temperature was increased to 920 °C, and the casting mold was held at this temperature for 2 h. An induction casting machine, Silvercast (Pi Dental, Budapest, Hungary), which utilizes electromagnetic induction and an electric spin, was used to cast the Co-Cr alloy samples.

L-PBF samples were fabricated using an DMG MORI Lasertec 12 SLM (DMG Mori Seiki, Bielefeld, Germany) with longitudinal axes inclined from the build direction by 45°. The parameters of the L-PBF sample printing process were as follows: laser power, 120 W; scanning velocity, 0.5 m/s; focus diameter, 39.5 µm; distance between two consequent laser scans (h), 0.051 mm; and layer thickness (d), 25 µm. DMG MORI Lasertec 12 SLM uses application-specific fiber lasers. Laser energy density (LED) was calculated as the ratio of laser power (P) to the product of scanning velocity (v), distance between two consecutive laser scans (h), and layer thickness (d). LED was 188 [J/mm^3^] and fell within the optimal range [12]. After fabrication, the samples were cut off and subjected to selected heat treatment.

### 2.2. Heat Treatment Parameters

Four different heat treatments (Table 2) were carried out under an argon gas environment in a muffle furnace (SNOL 4/12000, SnolTherm, Utena, Lithuania) only for the laser powder bed fusion Starbond alloy. Samples made using lost wax casting were not subjected to heat treatment.

Heating at 1150 °C for 1 h is sufficient for the molten pool boundaries disappearance and the formation of a homogeneous microstructure in L-PBF samples [21]. Therefore, all samples were placed in a heated oven (1150 °C) and kept there for 1 h.

Quenching of Co-Cr-based alloys causes the formation of small precipitations, and phase transformation occurs from the face-centered cubic (FCC, γ) phase to the hexagonal close-packed phase (HCP, the martensite ε) [31,32]. Water is the most common medium in quenching; however, the use of oil will reduce the cooling rate due to various factors (thermal conductivity, viscosity, density). To the best of the authors’ knowledge, such a quenching medium has not been used in research on dental Co-Cr alloys. Some of the samples, following homogenization heat treatment (at 1150 °C for 1 h), were quenched in either water (Water quenching) or oil (Oil quenching) to room temperature. The OH-70 oil (ORLEN OIL, Elbląg, Poland) was used.

Additional heating after quenching showed a positive effect on the strength properties through the formation of evenly distributed fine precipitates in the metal matrix [29]. After quenching in water or oil, certain samples underwent an additional heat treatment process (water quenching + reheating; oil quenching + reheating). The samples were reheated from room temperature to 750 °C, held at the maximum temperature for one hour, and then allowed to cool slowly in the furnace to room temperature.

### 2.3. Tensile Strength

The tensile test was performed using a universal testing machine (Servo LFV 50 kN; Walter-Bai, Switzerland) at a crosshead speed of 2 mm/min. The 0.2% yield strength (YP), ultimate tensile strength (FS), and Young’s modulus (E) were measured for the tensile test. The modulus (Ef) was calculated by software from the strain–stress curve. For each study group, 7 samples were tested. The fractures of selected samples were analyzed using a light microscope (Olympus BX 51, Tokyo, Japan).

### 2.4. Hardness

A hardness testing machine (Zwick, Germany) was used for the Vickers hardness measurements. A load of 1 kg was applied for 10 s of dwelling time. Nine measurements were made for each study group.

### 2.5. Sample Microstructure Characterization

The alloy microstructure was characterized by scanning electron microscopy combined with energy-dispersive X-ray spectroscopy (SEM-EDS).

After heat treatment, the rectangular samples were sequentially polished (abrasive papers up to 1000 grit), a 9-μm, and a 0.04-μm diamond suspension, followed by electropolishing in a solution of H_2_SO_4_/CH_3_OH (5:95) at 16–20 V and 268–273 K. Subsequently, the microstructures were investigated by scanning electron microscopy combined with energy dispersive X-ray spectroscopy under an accelerating voltage of 20 kV (JSM 6610LV, JEOL Ltd., Tokyo, Japan). Spot analyses were conducted to examine the compositional segregation in the samples by SEM-EDS. The working distance was set between 10–12 mm to obtain the most optimum acquisition parameters.

### 2.6. X-ray Diffraction Analysis

Phase identification was performed by X-ray diffraction (XRD; Panalytical Empyrean, Almeo, The Netherlands) using Cu Kα radiation at 40 kV and 40 mA and 2θ scans between 30° and 100°. The limited range of 2θ between 46° and 55° was selected for hexagonal close-packed (HCP) phase percentage calculations. In all the cases, the following configuration was used: parallel beam for Cu radiation, mask 10 mm, Soller slits 0.04 rad, divergence slit for mirror ½° mounted on the incident beam path, parallel plate collimator, Soller slits 0.04 rad, and proportional Xe detector mounted on the diffracted beam path.

The hexagonal close-packed phase content was calculated using three methods: the Chung method (semiquantitative) [33], the Rietveld method, which is a standard calculation method implemented in the XRD software package (High ScorePlus ver. 3.0e, Panalytical) module for Rietveld refinement, and the Sage–Guillaud method [34].

The fundamental problem in quantitative analysis lies in the mass absorption coefficient for the sample, (μ/ρ)s. If this quantity is known, the calculations are simple. The problem is that in most experiments (μ/ρ)s is a function of the amounts of the constituent phases.

From the internal standard equation a plot of $x_{\beta }\left(\frac{I_{\left(hkl\right)\alpha }}{I_{\left(hkl\right)\beta }}\right)$ vs. X_α_ (where X_α_ and X_β_ are the weight fractions of phases and I are the intensities) will be a straight line with slope *k*. Those *k* values using corundum as the β phase in a 50:50 mixture with the α phase are now published for many phases in the ICDD Powder Diffraction file, where *I*_(*hkl*)_ is defined as the 100% line for both phases. In the PDF “card” this is defined as *I*/*Ic*, the reference intensity ratio (RIR) for a 50:50 mixture of phase α and corundum.

Chung [33] recognized that if all phases in a mixture are known and if RIRs are known for all of those phases, then the sum of all of the fractions of all the phases must equal one. This allows the writing of a system of n equations to solve for the n weight fractions. Chung referred to this method as the matrix flushing or adiabatic principle, but it is now almost universally referred to as the normalized RIR method and allows “quantitative” calculations without the presence of an internal standard. It should be noted that the presence of any unidentified or amorphous phases invalidates the use of this method. It should be further noted that there can be undetectable phases in the sample; thus, the method will never rigorously work. Also, when peaks overlap, the main problem lies in their deconvolution.

In this work, the normalized method was used. This method assumes that the sum of all identified phases is 100%. This means there are neither unidentified crystalline phases nor an amorphous phase present in the sample. Only under these conditions can meaningful semi-quantitative results be obtained. The scale factor gives the relative intensity of each phase. By definition, the measured intensity of the strongest line of each phase should be used to calculate the scale factor. However, in HighScore software ver. 3.0e, Panalytical, the scale factor is determined by a least-squares fit through all matching reference pattern lines. This counteracts texture effects to a certain extent but does not follow the original definition.

In the Rietveld method, an observed and a calculated powder diffraction pattern are compared, and the difference is used to refine the atomic coordinates of the structure model. The calculated intensity at point *x_i_* is given by the equation.

y_cal_(*x_i_*) = s∑_k_m_k_(Lp)_k_|F_k_|^2^P_k_Φ(*x_i_* − *x_k_*) + b(*x_i_*)

where s is a scale factor, m_k_ is the reflection multiplicity, and P_k_ is a function to deal with the preferred orientation of the crystallites. They are two groups of refined parameters arising from the structure model (atomic coordinates x_j_, y_j_, and z_j_, atomic displacement parameters, unit-cell dimensions) and the instrumental model (angular dependence of the profile parameters, FWHM, and shape factors, 2θ-zero position, preferred orientation, etc.).

The precision of a structure refinement depends on many factors, e.g., data quality: counting statistics, texture, and line broadening. Moreover, structure refinement from X-ray diffraction data are strongly influenced by the fall-off of the scattering atomic factors with 2θ.

This paper presents the calculation results; peak shapes were described using the Pearson VII function, the background was determined manually, and crystallographic data were taken from the ICDD PDF 4+ database based on the phases identified by standard procedure using HighScore Plus software ver. 3.0e, Panalytical.

The relative amounts of FCC γ-Co and HCP ε-Co phases were predicted by measuring the integrated intensities of the FCC (200) and HCP (101) peaks. The weight fraction of the HCP ε-Co phase was calculated according to the following equation, developed by Sage and Guillaud.

$$f^{HCP}\left(wt\%\right)=\frac{I_{101}^{HCP}}{I_{101}^{HCP}+1.5\times I_{200}^{FCC}}$$

where f^HCP^ is the fraction of ε-HCP, and *I* is the integrated intensity of the corresponding XRD peaks.

The presented paper uses this equation as it can be found in the literature as widely applied in the case of examined alloys. However, this method does not consider any of the limiting parameters mentioned above for the Rietveld and RIR Chung methods.

### 2.7. Statistical Analysis

Statistical analyses were performed on the tensile strength and hardness data. Statistica 13.1 (StatSoft, Kraków, Poland) was used for the data analysis. Descriptive statistics were utilized for statistical analysis. The Shapiro–Wilk test was used to verify normality, while the Levene test was used to assess the homogeneity of variance. Because the assumptions of parametric tests were not met, the data were analyzed using the Kruskal–Wallis test with multiple comparisons of mean ranks. The accepted level of significance was set at α = 0.05. The accepted level of significance was α = 0.05.

## 3. Results

### 3.1. Tensile Strength

Table 3 summarizes the mechanical properties of the tested group. The cast samples exhibited the lowest tensile strength values, with Starbond at 707 MPa and MoguCera at 791 MPa. The highest tensile strength of 1389 MPa was observed for the samples subjected to the water quenching and reheating methods. The lowest elastic modulus values were recorded at 49 GPa for the samples subjected to water quenching, whereas the highest values of 127 GPa were found for the samples subjected to oil quenching combined with reheating.

The fractures of selected samples after completing the tensile test were analyzed, and fractography images were added in Appendix A (Figure A2(A–G)). The tensile fractures show intergranular character. It appears that samples have undergone failure mostly through brittle fracture mode with no obvious necking phenomenon around the fracture observed.

### 3.2. Hardness

The hardness results determined by the Vickers method are presented in Table 4.

The highest Vickers hardness was observed for water quenching and reheating (542 HV) and oil quenching and reheating (535 HV). The lowest HVs were observed for the MoguCera (345) and Starbond (330) groups (Table 4).

### 3.3. Sample Microstructure Characterization

**Figure 1:**
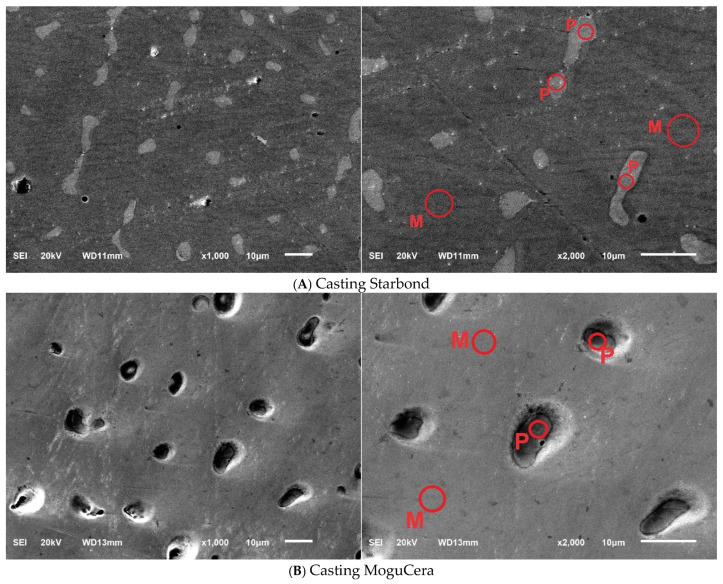

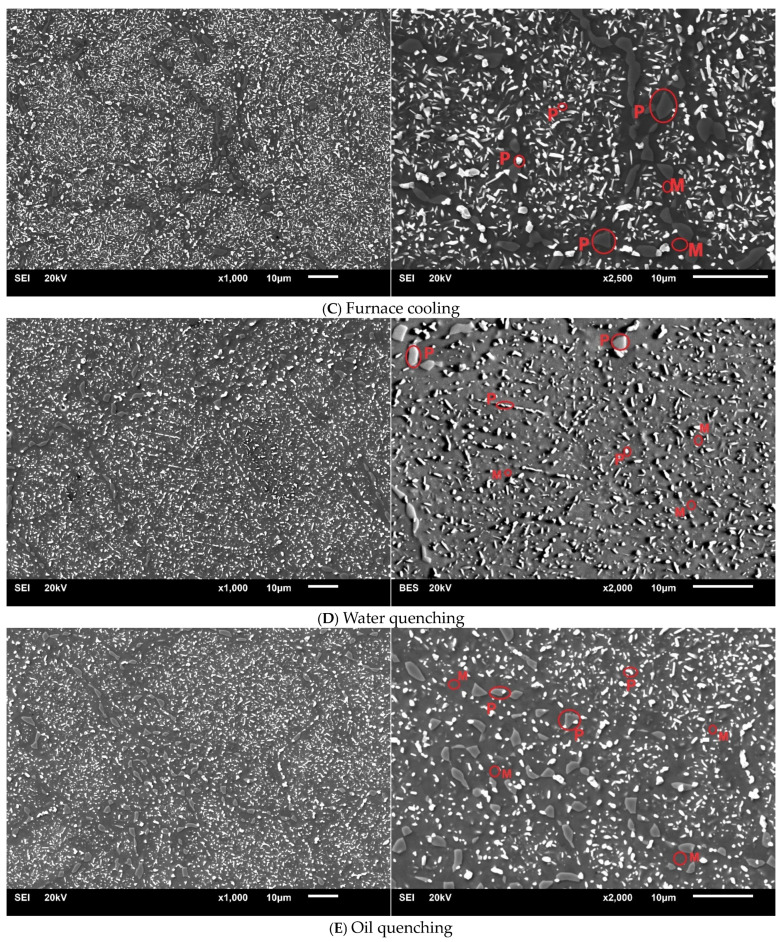

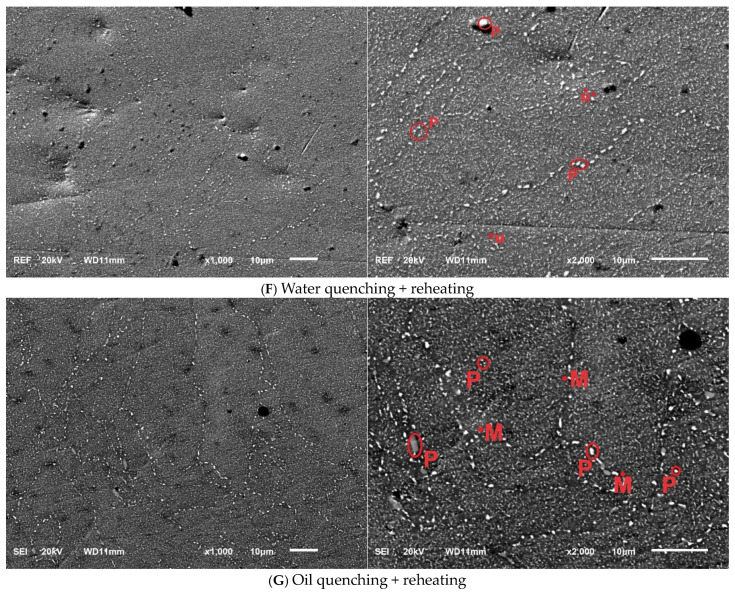
Scanning electron microscopy (SEM) micrograph of Co-Cr alloys at 1000× and 2000× magnification with matrix (M) and participation (P) labeling (**A**) Starbond COS cast samples, (**B**) MoguCera cast samples, (**C**–**G**) Starbond COS samples produced by laser powder bed fusion after heat treatments: (**C**) heat treatment at 1150 °C and furnace cooling, (**D**) heat treatment at 1150 °C and cooling in water, (**E**) heat treatment at 1150 °C and cooling in oil, (**F**) heat treatment at 1150 °C, cooling in water, and reheating at 750 °C, (**G**) Heat treatment at 1150 °C, cooling in oil, and reheating at 750 °C.

Selected SEM micrographs showing the surface of the Co-Cr samples are presented in Figure 1A–G.

The microstructures of the tested samples differ from each other. Large precipitates are visible in the Starbond and MoguCera casts (Figure 1A,B). After water quenching followed by reheating (Figure 1F) and oil quenching followed by reheating (Figure 1G), the samples are characterized by having the most homogeneous structure with small precipitates both inside the grains and on their borders. The presented in Figure 1 SEM images (2000× magnification) illustrate the presence of Co-matrix with precipitates. The average percentages of elements for the studied groups are presented in Table 5. EDS measurements (matrix (1), large precipitate (2), smaller precipitate (3)) show that the solid solution matrix consists mainly of Co (>60%) and Cr (15–27%), while the precipitates contain larger amounts of W and Mo.

The XRD patterns of the tested samples are presented in Appendix A (Figure A3, Figure A4, Figure A5, Figure A6, Figure A7, Figure A8 and Figure A9). The face-centered cubic (FCC) cobalt phase was detected in almost all samples. In L-PBF samples, there are signals representing various interphase precipitates. Based on the obtained X-ray profiles, the hexagonal close-packed (HCP) phase volume fractions were calculated using the semiquantitative (Chung), Rietveld, and Sage-Guillaud methods. The concentration of the HCP phase is presented in Table 6.

The volume fractions of the HCP slightly differ among the employed calculation methods. The highest concentration was observed for the MoguCera Cast sample (82–86%), and the lowest was observed for the water-quenched sample (0%).

## 4. Discussion

In parts produced by laser powder bed fusion, residual stresses occur due to rapid thermal changes in melting and cooling during the fabrication process [23]. This may lead to microcracking or distortion, which can result in treatment failure. Therefore, post-heat treatments are crucial for enhancing the mechanical characteristics of components manufactured through L-PBF. Standard processing used after product manufacturing includes heating and cooling in an oven [23,35]. However, to increase the strength and improve the wear resistance of Co-Cr alloys, certain treatments can be carried out to increase the volume of the hexagonal close-packed (HCP) phase in addition to the face-centered cubic (FCC) phase [36]. The FCC → HCP transformation can be induced athermally (by quenching), isothermally (annealing between 650 °C and 950 °C), or through plastic strain [37,38]. The phase transformation from FCC to HCP in Co-Cr alloys is influenced by factors such as composition, temperature, and residual stress. CoCrMo alloys undergo complex phase changes, including martensite, massive transformation, discontinuous precipitation, eutectoid reaction, and secondary phase precipitation [34], during heat treatment. The key element was the carbon content. It stabilizes the γ-FCC form, affects the transformation kinetics, promotes carbide precipitation, and suppresses the σ-phase. Cr and Mo stabilize ε-HCPs by reducing the stacking fault energy of pure cobalt [26]. Due to the lack of information on the exact elemental composition of commercial materials (trade know-how) and increasing popularity of modern technologies in dental techniques, providing insights into the behavior of CoCrMo alloys after specific heat treatments is very important in dental research.

In this study, the effects of a high cooling rate of heat-treated samples (cooling in water/oil) and additional annealing after quenching were evaluated for Co-Cr dental alloys. The groups were compared to those cooled in the furnace and cast samples (standard manufacturing method). Based on the data presented above, the null hypothesis must be rejected, as significant dissimilarities were found among the L-PBF groups. The second hypothesis can be rejected due to differences in properties between Co-Cr samples prepared with the lost wax and those obtained by L-PBF.

The properties of Co-Cr-Mo alloys depend on many factors, such as the microstructure, morphology, composition, FCC-HCP ratio, carbides, and intermetallic precipitates. In addition, properties are also affected by phase, grain orientation, grain boundary conditions, defects, etc. These properties are influenced by all the steps taken to produce the final product [39,40,41]. One of the most important elements is the selection of manufacturing technology and its parameters. Our results (Table 3) are consistent with other studies, where the materials obtained by L-PBF, even before thermal treatment, were characterized by higher tensile strength and 0.2 yield point values than the samples obtained by the traditional casting method [42,43]. The Vickers hardness of the cast samples (Starbond and MoguCera) was lower than that of the L-PBF samples (Table 4). These findings are consistent with those of previous studies [44,45,46]. The tensile strength and 0.2% yield point values for materials produced using the L-PBF method exceed 1000 MPa and 750 MPa, respectively, while for cast samples, these values do not surpass 780 MPa and 590 MPa. In terms of hardness, the 3D-printed samples exhibit values above 400 HV, which is significantly higher than the 300 HV observed for materials produced using traditional casting methods [40,47,48]. The cast Co–Cr alloys exhibit high porosity, which is related to the lack of compensated shrinkage during solidification (the formation of a dendritic structure). In contrast, a nearly perfect alloy density can be obtained by L-PBF technology when proper manufacturing parameters are selected. Then, complete melting and rapid cooling processes occur in the very small local area, resulting in minimal porosity [12,49]. The microstructure is another factor contributing to the observed differences in properties between the traditional method and the L-PBF method. L-PBF materials are characterized by a finer microstructure, with smaller intermetallic compounds precipitated in the Co-matrix across all heat treatment groups (Figure 1). In addition, Mogucera and Starbond COS alloys show some dendric microstructure, which can be observed in cast samples of Co-Cr alloys [50]. It was shown that the hardness of the dendric is lower than the microhardness of the alloy and intra-dendric regions [51]. Finer microstructure, small precipitations, and no visible dendric microstructure may explain why the mechanical properties (FS, HV) of the L-PBF-fabricated samples were better than those of the cast samples. Laser-sintered materials are more consistent and homogeneous [52,53]. Therefore, manufacturing dental prostheses using the L-PBF method prevents unexpected failures due to porosity, ensuring a more predictable treatment outcome [49].

Another element affecting the material properties of the final metallic product is heat treatment. Despite the advantages of the L-PBF process over casting (labor efficiency, cost savings, precision, very low porosity, good mechanical properties), these materials should undergo heat treatment, which allows them to achieve a homogeneous structure and eliminate residual stresses [18,54,55]. It was shown that selected heat treatments of the L-PBF sample affect the tensile strength (Table 3), which agrees with other studies [56,57]. The TS values and 0.2% YS are greater after the proposed heat treatments than those for the as-built Co-Cr (1173 MPa (TS), 839 MPa (0.2% YS), and 12.3% elongation) [23]. The amounts, morphologies, and distributions of precipitates are crucial for the mechanical properties of a material. The slower cooling in the furnace allowed more time for elemental diffusion and the formation of larger precipitates at the grain boundaries. A similar effect can be observed in oil (Figure 1C,E). The finest microstructure was found in the water-quenched sample. Given the chromium content exceeding 10%, these carbides could be M6C. These specified carbides exhibit high brittleness and possess a crystal structure characterized by an asymmetric hexagonal cell [29,58]. These precipitates, which were formed greatly at the grain boundaries and in the matrix, reduced Young’s modulus and brittle fracturing for samples cooled in a furnace or quenched in water and oil (Table 3). Some precipitates occurred in straight lines and along the crystal grain boundaries (Figure 1F,G). The emergence of ε-martensite (HCP) manifests in the form of elongated straight lines, where carbides precipitate during the reheating process. Precipitates improve the overall mechanical, abrasive, and corrosion properties of Co-Cr alloys [44]. Precipitates impede dislocation slip, enhancing strength but reducing ductility. The strengthening effect increases as precipitate spacing decreases, meaning more numerous and smaller precipitates enhance strength [57]. The best properties were achieved for samples subjected to additional annealing after quenching. They were characterized by a homogeneous microstructure with precipitates present both at grain boundaries and within the matrix (Figure 1F,G, Table 5).

The XRD analysis indicated the existence of a cubic phase (FCC) of Co–Cr and a hexagonal phase (HCP) of Co–Cr in almost every tested material. The exceptions were the quenched samples in water (Table 6, Figure A3, Figure A4, Figure A5, Figure A6, Figure A7, Figure A8 and Figure A9). In the XRD results (Figure A3, Figure A4, Figure A5, Figure A6, Figure A7, Figure A8 and Figure A9), numerous phase signals can be observed. The most prominent signal in all the examined groups originated from the FCC phase. The percentage content of the HCP phase was within the range of 0–86%. Unfortunately, the XRD method does not allow for determining the exact compositions of the remaining signals due to the influence of W and Mo on the formation of various precipitates [13,59]. These signals represent various interphase precipitates. The highest amount of HCP was observed for the MoguCera material (average 85%, Table 6), which may be primarily due to the difference in the composition of the material (lack of W). In the cast samples of Starbond COS, tungsten became part of the precipitate, as was observed in the XRD patterns (Figure A3). When comparing L-PBF materials, the highest values were for materials subjected to additional heating. It was shown that HCP phased transformation by athermal processes (quenching) is limited and does not proceed beyond a volume fraction of 0.4–0.5. Using isothermal aging at 650–950 °C, a complete transformation of the FCC phase to the HCP phase is possible [28]. Although the HCP phase should be created during quenching, the tungsten and carbon content may also stabilize the FCC phase for cobalt-based alloys, which influences the disturbance of the athermal martensitic transformation during rapid cooling [58]. Materials subjected to the annealing process are characterized by a high modulus of elasticity. This is consistent with the observations of other researchers showing that the HCP phase improves strength, hardness, and wear resistance [60].

**Figure A1:**
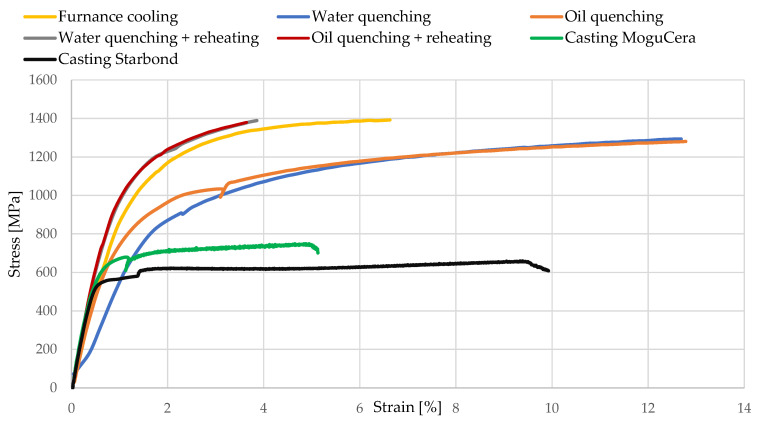
Stress-strain curves of tensile test specimens: Starbond COS cast samples, MoguCera cast samples, and Starbond COS samples produced by laser powder bed fusion after heat treatments: Furnace cooling—heat treatment at 1150 °C and furnace cooling; Water quenching—heat treatment at 1150 °C and cooling in water; Oil quenching—heat treatment at 1150 °C and cooling in oil; Water quenching + reheating—heat treatment at 1150 °C, cooling in water, and reheating at 750 °C; Oil quenching + reheating—Heat treatment at 1150 °C, cooling in oil, and reheating at 750 °C.

**Figure A2:**
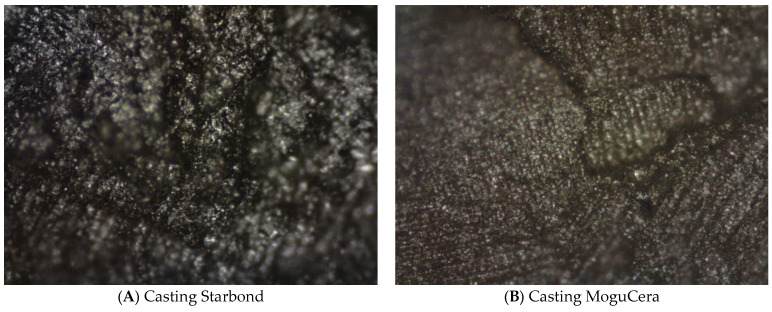

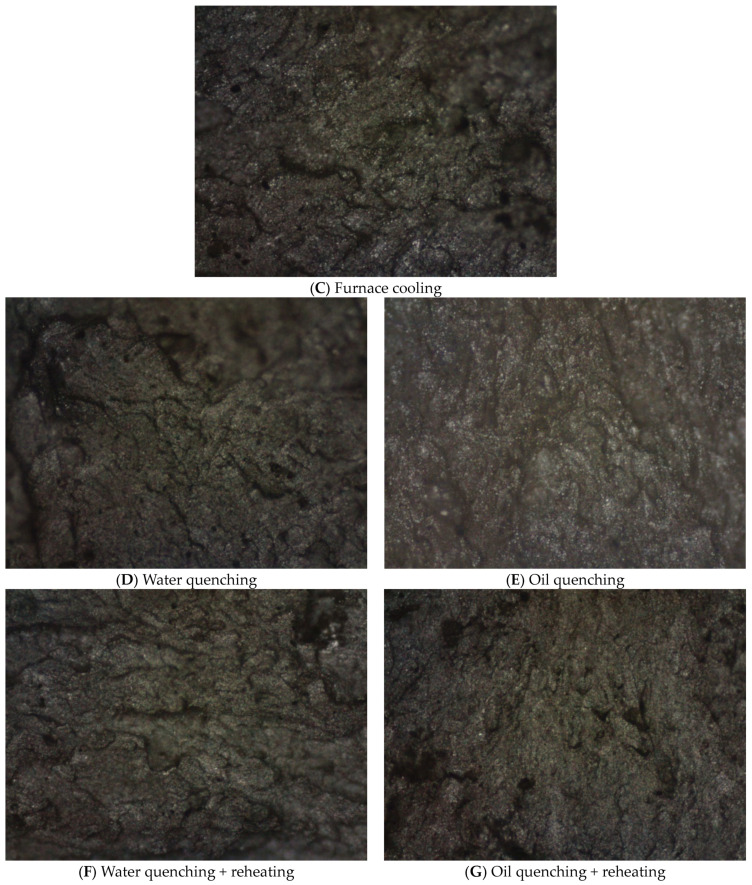
Fractures of samples after tensile tests of Co-Cr alloys at × 50 magnification. (**A**) Starbond COS cast samples, (**B**) MoguCera cast samples, (**C**–**G**) Starbond COS samples produced by laser powder bed fusion after heat treatments: (**C**) heat treatment at 1150 °C and furnace cooling, (**D**) heat treatment at 1150 °C and cooling in water, (**E**) heat treatment at 1150 °C and cooling in oil, (**F**) heat treatment at 1150 °C, cooling in water, and reheating at 750 °C, (**G**) Heat treatment at 1150 °C, cooling in oil, and reheating at 750 °C.

**Figure A3:**
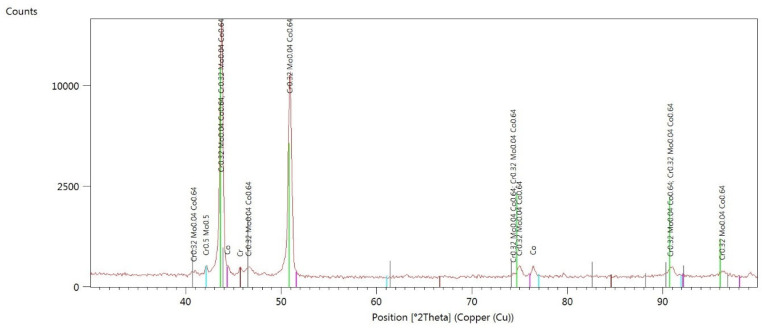
X-ray diffraction (XRD) patterns of casting Starbond sample.

**Figure A4:**
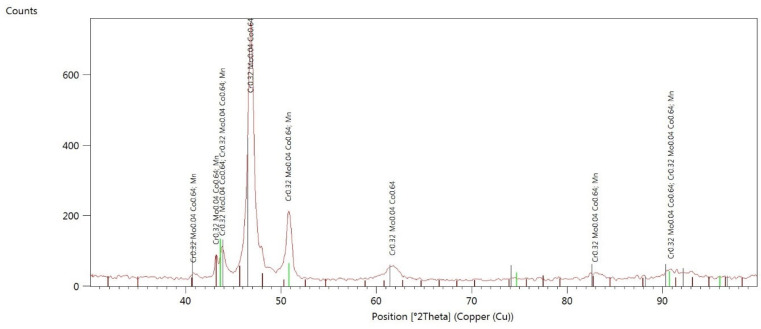
X-ray diffraction (XRD) patterns of casting MoguCera sample.

**Figure A5:**
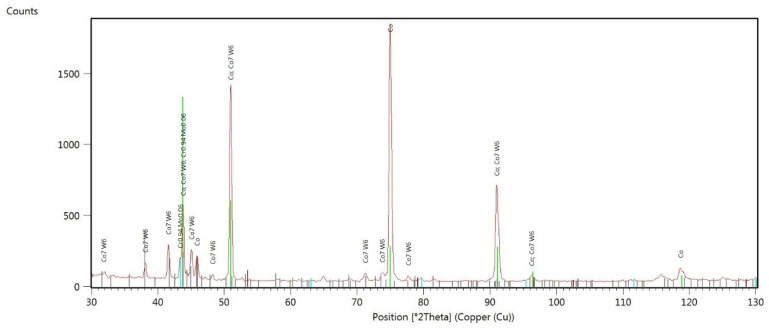
X-ray diffraction (XRD) patterns of furnace cooling sample.

**Figure A6:**
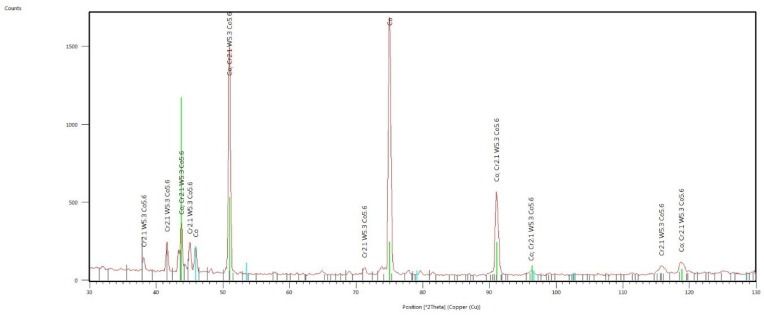
X-ray diffraction (XRD) patterns of water quenching sample.

**Figure A7:**
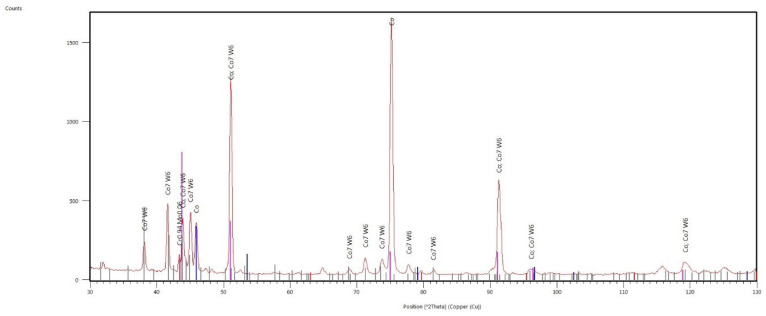
X-ray diffraction (XRD) patterns of oil quenching sample.

**Figure A8:**
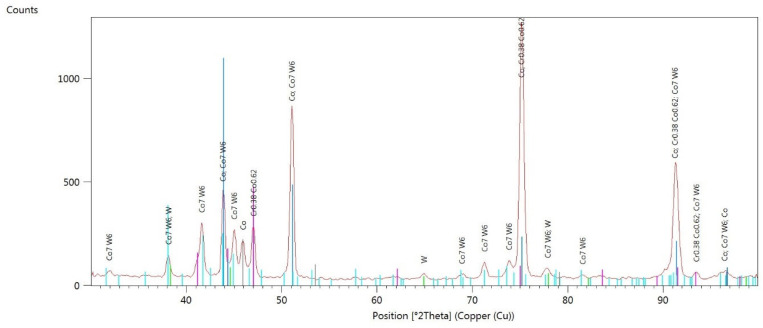
X-ray diffraction (XRD) patterns of water quenching followed by reheating sample.

**Figure A9:**
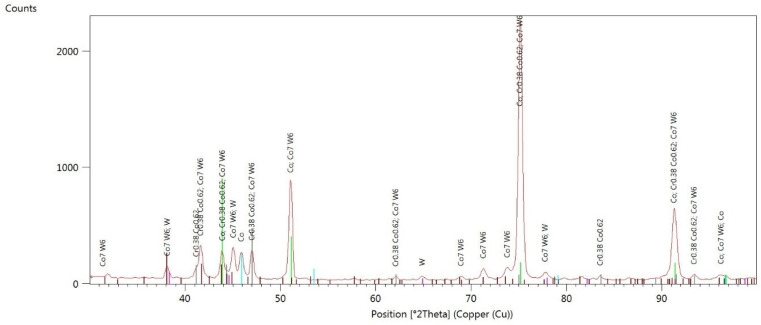
X-ray diffraction (XRD) patterns of oil quenching followed by reheating sample.

Fast cooling (water quenching) caused the samples to be characterized by the lowest Young’s modulus (49 GPa) than that of other research groups (93–127 GPa). The highest values were achieved for the samples treated with oil quenching and reheating. The fracture surface predominantly exhibits smooth cleavage planes, indicative of typical brittle fracture behavior associated with low ductility, although some small and shallow dimples are also present (Figure A2), which is in agreement with other studies [31]. Lower values of the modulus of elasticity may positively affect the performance properties of dental clasps due to the more flexible nature of the material. In other studies, Young’s modulus was greater than 200 GPa [42,47]. Some difference may result from the inaccuracy of our measurement method—lack of an extensometer. It has been demonstrated that extensometers should be prioritized for determining Young’s modulus, followed by the use of strain gauges. Unfortunately, utilizing the machine crosshead motion is not recommended due to the high variability in strain measurements [61]. Figure A1 presents representative stress-strain curves, highlighting the variations observed among the studied groups. While these findings may differ from those reported in other studies, they remain comparable and effectively illustrate the differences between the experimental groups. Additionally, it is worth noting that the values of the modulus of elasticity are often not reported in studies investigating the impact of heat treatment on the properties of Co-Cr alloys produced by the L-PBF method [39,57]. Hegele et al. [62] and von Kobylinski et al. [63] suggest that changes in the modulus of elasticity may be more dependent on small macroscopic residual stress levels present in the material. Considering the quenching method used in this study, it not only induces differences in the microstructure but also may generate residual thermal stresses in the quenched material [64], which according to von Kobylinski et al. may result in a decrease in the modulus of elasticity. Conversely, subsequent annealing and slow cooling reduce these stresses [65,66], thereby increasing the modulus of elasticity. However, there is a need for further research on the link between residual stresses and Young’s modulus, especially after different heat treatments.

The obtained results show that products manufactured using the traditional method (lost wax casting) have worse mechanical properties due to the coarser microstructure, porosity, presence of larger precipitates, and the visible dendritic microstructure. Additionally, this method is time-consuming and costly (many different materials are used for production). Thus, while L-PBF techniques represent the future of dentistry, there remains a need for further development of optimized heat treatment protocols to fully enhance their performance. Athermal (quenching in water/oil) and isothermal (annealing process) heat treatment of L-PBF samples results in different microstructures (Figure 1D–G) and phase volume fractions (Table 6) affecting their mechanical properties (Table 3). The quenching results in the formation of a high amount of brittle M6C precipitates at grain boundaries, resulting in lower mechanical properties due to the brittleness of these carbides [29]. The transformation to HCP is limited during athermal quenching processes but can be more fully achieved through isothermal aging [28]. The HCP fraction (~16% average) results in greater mechanical properties due to the atomic structure of this phase and the restriction of the slip in the fcc phase in the presence of the hcp [67].

Considering the use of the tested materials as clasps, the next stage will be to verify whether the proposed thermal treatment methods improve fatigue properties.

## 5. Conclusions

The influence of heat treatment on the tensile strength, hardness, and microstructure of Co-Cr laser-melted samples was investigated. The resulting properties were also compared with those of as-cast alloys. Based on the results and taking into account the limitations of this study, the following conclusions can be drawn:

1. The microstructures and mechanical properties of Co-Cr dental alloys are dependent on the manufacturing method.
2. The microstructure, distribution of precipitates, face-centered cubic (FCC) phase, and hexagonal close-packed (HCP) phase determined the final properties of the Co-Cr samples.
3. The mechanical properties (flexural strength and hardness) of the laser powder bed fusion (L-PBF) specimens were greater than those of the samples obtained by the lost wax casting method.
4. Annealing after quenching results in a more homogenous microstructure with a fine distribution of precipitates inside grains and on their borders.
5. The L-PBF specimens mainly consisted of the FCC phase. The highest HCP values (average of approximately 16%) were obtained for samples treated with additional heat treatment after quenching.

## Figures and Tables

**Table 1:** Nominal chemical composition (in wt. %) of the used Co–Cr alloys provided by manufacturers.

Materials	Production Method	Manufacturer	Composition
Starbond COS powder	laser powder bed fusion	Scheftner, Mainz, Germany	Co 59%, Cr 25%, W 9.5%, Mo 3.5%, Si 1%, C, Fe, Mn, N < 1%
Starbond COS ingots	casting	Scheftner, Mainz, Germany	Co 59%, Cr 25%, W 9.5%, Mo 3.5%, Si 1%, C, Fe, Mn, N < 1%
MoguCera C ingots	casting	Scheftner, Mainz, Germany	Co 65%, Cr 28%, Mo 5%, Mn 1%, C, Si < 1%

Co—cobalt, Cr—chromium, W—tungsten, Mo—molybdenum, Si—silicon, C—carbon, Fe—iron, Mn—manganese, N—nitrogen.

**Table 2:** Experimental plan for the investigations of heat treatment conditions of additively manufactured specimens.

Production Method	Group	Heat Treatment Conditions	Assumption
Casting	Casting Starbond	none	For casting samples, there was no post-production heat treatment after cooling at room temperature; control.
	Casting MoguCera	none	
Laser powder bed fusion	Furnace cooling	Heat treatment at 1150 °C for one hour, cooling slowly to room temperature in a furnace	The heat treatment to homogenize the alloy microstructure; control.
	Water quenching	Heat treatment at 1150 °C, one hour, and water quenching	The martensitic HCP phase in Co-Cr-Mo-C alloys and small precipitates occurrence to improve the mechanical strength of the alloy.
	Oil quenching	Heat treatment at 1150 °C, one hour, and oil quenching (OH-70, ORLEN OIL, Elbląg, Poland)	The martensitic HCP phase in Co-Cr-Mo-C alloys and small precipitates occurrence to improve the mechanical strength of the alloy.
	Water quenching + reheating	Heat treatment at 1150 °C for one hour and water quenching; after that, the samples were reheated for 1 h at 750 °C	The quenching is followed by a reheating to improve the creep strength of the alloy.
	Oil quenching + reheating	Heat treatment at 1150 °C for one hour and oil quenching; after that, the samples were reheated for 1 h at 750 °C	The quenching is followed by a reheating to improve the creep strength of the alloy.

HCP—hexagonal close-packed phase.

**Table 3:** Mechanical properties (TS—tensile strength, E—elastic modulus, 0.2 YP—0.2% yield strength) of Co-Cr specimens obtained by casting and laser powder bed fusion, which were conducted with different heat treatments.

Group	TS [MPa]	E [GPa]	0.2 YP [MPa]
Casting Starbond	707 (58) ^a,b,c^	100 (13)	525 (11) ^a,b,c^
Casting MoguCera	791 (67) ^d,e^	114 (7) ^a,d^	599 (52) ^d,e,f^
Furnace cooling	1366 (37) ^a,d^	90 (3) ^a,b,c^	909 (25) ^a,d^
Water quenching	1267 (41)	49 (8) ^d,e,f^	706 (40)
Oil quenching	1298 (52)	93 (9) ^g^	787 (54)
Water quenching + reheating	1389 (38) ^b,e^	116 (13) ^b,e^	882 (140) ^b.e^
Oil quenching + reheating	1368 (142) ^c^	127 (17) ^c,f,g^	876 (106) ^c,f^

Statistical differences are presented within individual research methods (columns); the results with the same assigned letter show a statistical difference at the level of *p* ≤ 0.05.

**Table 4:** Vickers hardness (HV) of Co-Cr specimens obtained by casting and laser powder bed fusion, which were conducted with different heat treatments.

Group	HV [MPa]
Casting Starbond	330 (41) ^a,b,c^
Casting MoguCera	345 (39) ^d,e,f^
Furnace cooling	526 (28) ^a,d^
Water quenching	472 (21) ^g^
Oil quenching	475 (37)
Water quenching + reheating	542 (11) ^b,e,g^
Oil quenching + reheating	535 (18) ^c,f^

Statistical differences are presented within individual research methods (columns); the results with the same assigned letter show a statistical difference at the level of *p* ≤ 0.05.

**Table 5:** Average weight percentages of chemical elements determined on the basis of EDS measurements in three different areas (matrix, large precipitates, and smaller precipitates) for these study groups.

Sample	Matrix	Large Precipitates	Smaller Precipitates
Starbond Cast	Co 61%	Co 44%	Co 45%
	Cr 15%	Cr 29%	Cr 22%
	W 9%	W 17%	W 18%
	Mo 3%	Mo 9%	Mo 13%
MoguCera Cast	Co 65%	Co 49%	Co 48%
	Cr 28%	Cr 28%	Cr 28%
	Mo 4.8%	Mo 16%	Mo 16%
Furnace cooling	Co 61%	Co 53%	Co 59%
	Cr 26%	Cr 26%	Cr 26%
	W 9.5%	W 14%	W 10%
	Mo 4%	Mo 6%	Mo 4%
Water quenching	Co 62%	Co 46%	Co 42%
	Cr 27%	Cr 26%	Cr 28%
	W 7%	W 19%	W 19%
	Mo 4%	Mo 9%	Mo 11%
Oil quenching	Co 62%	Co 44%	Co 42%
	Cr 27%	Cr 25%	Cr 19%
	W 8%	W 21%	W 28%
	Mo 4%	Mo 10%	Mo 11%
Water quenching + reheating	Co 61%	Co 42%	Co 53%
	Cr 27%	Cr 19%	Cr 24%
	W 8%	W 28%	W 16%
	Mo 3%	Mo 10%	Mo 6%
Oil quenching + reheating	Co 55%	Co 43%	Co 47%
	Cr 24%	Cr 22%	Cr 21%
	W 16%	W 26%	W 23%
	Mo 5%	Mo 9%	Mo 8%

Co—cobalt, Cr—chromium, W—tungsten, Mo—molybdenum.

**Table 6:** Hexagonal close-packed phase concentration calculated by employing the Chung (semi-quantitative), Rietveld, Sage, and Guillaud methods for this study groups.

Sample	Chung	Rietveld	Sage-Guillaud
Starbond Cast	5	6.7	9.4
MoguCera Cast	86	82.0	86.0
Furnace cooling	1	0.3	0.5
Water quenching	0	0.0	0.0
Oil quenching	1	0.5	0.6
Water quenching + reheating	26	11.3	15.4
Oil quenching + reheating	20	7.8	11.6

## Data Availability

The original contributions presented in the study are included in the article, further inquiries can be directed to the corresponding author.

## References

[1] Fung L., Brisebois P. (2020). Implementing Digital Dentistry into Your Esthetic Dental Practice. Dent. Clin. N. Am..

[2] Papadiochou S., Pissiotis A.L. (2018). Marginal adaptation and CAD-CAM technology: A systematic review of restorative material and fabrication techniques. J. Prosthet. Dent..

[3] Haleem A., Javaid M. (2019). 3D scanning applications in medical field: A literature-based review. Clin. Epidemiol. Glob. Health.

[4] Javaid M., Haleem A., Kumar L. (2019). Current status and applications of 3D scanning in dentistry. Clin. Epidemiol. Glob. Health.

[5] Javaid M., Haleem A. (2019). Current status and applications of additive manufacturing in dentistry: A literature-based review. J. Oral Biol. Craniofacial Res..

[6] Strub J.R., Rekow E.D., Witkowski S. (2006). Computer-aided design and fabrication of dental restorations: Current systems and future possibilities. J. Am. Dent. Assoc..

[7] Liparoti S., Sofia D., Romano A., Marra F., Pantani R. (2021). Fused filament deposition of pla: The role of interlayer adhesion in the mechanical performances. Polymers.

[8] Ngo T.D., Kashani A., Imbalzano G., Nguyen K.T.Q., Hui D. (2018). Additive manufacturing (3D printing): A review of materials, methods, applications and challenges. Compos. Part B Eng..

[9] Revilla-León M., Özcan M. (2017). Additive Manufacturing Technologies Used for 3D Metal Printing in Dentistry. Curr. Oral Health Rep..

[10] Dikova T., Dobrzański L.A. (2018). Properties of Co-Cr Dental Alloys Fabricated Using Additive Technologies. Biomaterials in Regenerative Medicine.

[11] Wang J., Ren J., Liu W., Wu X., Gao M., Bai P. (2018). Effect of Selective Laser Melting Process Parameters on Microstructure and Properties of Co-Cr Alloy. Materials.

[12] Tonelli L., Fortunato A., Ceschini L. (2020). CoCr alloy processed by Selective Laser Melting (SLM): Effect of Laser Energy Density on microstructure, surface morphology, and hardness. J. Manuf. Process..

[13] Sun S.H., Koizumi Y., Kurosu S., Li Y.P., Matsumoto H., Chiba A. (2014). Build direction dependence of microstructure and high-temperature tensile property of Co-Cr-Mo alloy fabricated by electron beam melting. Acta Mater..

[14] Zhou X., Li K., Zhang D., Liu X., Ma J., Liu W., Shen Z. (2015). Textures formed in a CoCrMo alloy by selective laser melting. J. Alloys Compd. J..

[15] Gu D., Shi Q., Lin K., Xi L. (2018). Microstructure and performance evolution and underlying thermal mechanisms of Ni-based parts fabricated by selective laser melting. Addit. Manuf..

[16] Yap C.Y., Chua C.K., Dong Z.L., Liu Z.H., Zhang D.Q., Loh L.E., Sing S.L. (2015). Review of selective laser melting: Materials and applications. Appl. Phys. Rev..

[17] Béreš M., Silva C.C., Sarvezuk P.W.C., Wu L., Jardini A.L., Feitosa A.L.M., Žilková J., de Abreu H.F.G., Filho R.M. (2018). Mechanical and phase transformation behaviour of biomedical Co-Cr-Mo alloy fabricated by direct metal laser sintering. Mater. Sci. Eng. A.

[18] Takaichi A., Kajima Y., Kittikundecha N., Linn H., Htoot H., Cho W., Hanawa T., Yoneyama T., Wakabayashi N. (2020). Effect of heat treatment on the anisotropic microstructural and mechanical properties of Co–Cr–Mo alloys produced by selective laser melting. J. Mech. Behav. Biomed. Mater..

[19] Lu Y., Wu S., Gan Y., Li J., Zhao C., Zhuo D., Lin J. (2015). Investigation on the microstructure, mechanical property and corrosion behavior of the selective laser melted CoCrW alloy for dental application. Mater. Sci. Eng. C.

[20] Yan X., Lin H., Wu Y., Bai W. (2018). Effect of two heat treatments on mechanical properties of selective-laser-melted Co-Cr metal-ceramic alloys for application in thin removable partial dentures. J. Prosthet. Dent..

[21] Seki E., Kajima Y., Takaichi A., Kittikundecha N., Htoot H., Cho W., Linn H., Doi H., Hanawa T., Wakabayashi N. (2019). Effect of heat treatment on the microstructure and fatigue strength of CoCrMo alloys fabricated by selective laser melting. Mater. Lett..

[22] Konieczny B., Szczesio-Wlodarczyk A., Sokolowski J., Bociong K. (2020). Challenges of Co–Cr Alloy Additive Manufacturing Methods in Dentistry—The Current State of Knowledge (Systematic Review). Materials.

[23] Kajima Y., Takaichi A., Kittikundecha N., Nakamoto T., Kimura T., Nomura N., Kawasaki A., Hanawa T., Takahash H., Wakabayashi N. (2018). Effect of heat-treatment temperature on microstructures and mechanical properties of Co– Cr–Mo alloys fabricated by selective laser melting. Mater. Sci. Eng. A.

[24] Viderščak D., Schauperl Z., Šolić S., Ćatić A., Godec M., Kocijan A., Paulin I., Donik Č. (2021). Additively manufactured commercial Co-Cr dental alloys: Comparison of microstructure and mechanical properties. Materials.

[25] Hong M.H., Lee D.H., Hanawa T., Kwon T.Y. (2022). Comparison of microstructures and mechanical properties of 3 cobalt-chromium alloys fabricated with soft metal milling technology. J. Prosthet. Dent..

[26] López H.F., Saldivar-Garcia A.J. (2008). Martensitic transformation in a cast Co-Cr-Mo-C alloy. Metall. Mater. Trans. A Phys. Metall. Mater. Sci..

[27] Boyer H.E., Archambault P., Moreaux F., Kobasko N.I. (1992). Techniques of Quenching. Theory and Technology of Quenching.

[28] Turrubiates-Estrada R., Salinas-Rodriguez A., Lopez H.F. (2011). FCC to HCP transformation kinetics in a Co-27Cr-5Mo-0.23C alloy. J. Mater. Sci..

[29] Sedlaček M., Zupančič K., Šetina Batič B., Kosec B., Zorc M., Nagode A. (2023). Influence of Precipitation Hardening on the Mechanical Properties of Co-Cr-Mo and Co-Cr-W-Mo Dental Alloys. Metals.

[30] Sing S.L., Huang S., Yeong W.Y. (2020). Effect of solution heat treatment on microstructure and mechanical properties of laser powder bed fusion produced cobalt-28chromium-6molybdenum. Mater. Sci. Eng. A.

[31] Lu Y., Wu S., Gan Y., Zhang S., Guo S., Lin J., Lin J. (2016). Microstructure, mechanical property and metal release of As-SLM CoCrW alloy under different solution treatment conditions. J. Mech. Behav. Biomed. Mater..

[32] Khaimanee P., Choungthong P., Uthaisangsuk V. (2017). Effects of Isothermal Aging on Microstructure Evolution, Hardness and Wear Properties of Wrought Co-Cr-Mo Alloy. J. Mater. Eng. Perform..

[33] Chung F.H. (1974). Quantitative interpretation of X-ray diffraction patterns of mixtures. I. Matrix-flushing method for quantitative multicomponent analysis. J. Appl. Crystallogr..

[34] Donkor B.T., Song J., Fu Y., Kattoura M., Mannava S.R., Steiner M.A., Vasudevan V.K. (2020). Accelerated γ-face-centered cubic to ε-hexagonal close packed massive transformation in a laser powder bed fusion additively manufactured Co-29Cr-5Mo alloy. Scr. Mater..

[35] Htoot H., Cho W., Takaichi A., Kajima Y., Htat H.L., Kittikundecha N., Hanawa T., Wakabayashi N. (2021). Effect of Post-Heat Treatment Cooling Conditions on Microstructures and Fatigue Properties of Cobalt Chromium Molybdenum Alloy Fabricated through Selective Laser Melting. Metals.

[36] Kaita W., Hagihara K., Augusto L., Nakano T. (2018). Plastic deformation mechanisms of biomedical Co–Cr–Mo alloy single crystals with hexagonal close-packed structure. Scr. Mater..

[37] Lashgari H.R., Zangeneh S., Hasanabadi F., Saghafi M. (2010). Microstructural evolution during isothermal aging and strain-induced transformation followed by isothermal aging in Co-Cr-Mo-C alloy: A comparative study. Mater. Sci. Eng. A.

[38] Ramirez-Ledesma A.L., Lopez-Molina E., Lopez H.F., Juarez-Islas J.A. (2016). Athermal ε-martensite transformation in a Co-20Cr alloy: Effect of rapid solidification on plate nucleation. Acta Mater..

[39] Stamenković D., Popović M., Rudolf R., Zrilić M., Raić K., Đuričić K.O., Stamenković D. (2023). Comparative Study of the Microstructure and Properties of Cast-Fabricated and 3D-Printed Laser-Sintered Co–Cr Alloys for Removable Partial Denture Frameworks. Materials.

[40] Zhou Y., Li N., Yan J., Zeng Q. (2018). Comparative analysis of the microstructures and mechanical properties of Co-Cr dental alloys fabricated by different methods. J. Prosthet. Dent..

[41] Kassapidou M., Stenport V.F., Johansson C.B., Syverud M., Hammarström Johansson P., Börjesson J., Hjalmarsson L. (2023). Cobalt chromium alloys in fixed prosthodontics: Investigations of mechanical properties and microstructure. J. Prosthet. Dent..

[42] Alloys D., Cad T., Kim K., Kwon T. (2016). Microstructures and Mechanical Properties of Co-Cr Dental Alloys Fabricated by Three CAD/CAM-Based Processing Techniques. Materials.

[43] Koutsoukis T., Zinelis S., Eliades G., Al-wazzan K., Al Rifaiy M., Jabbari Y.S. (2015). Al Selective Laser Melting Technique of Co-Cr Dental Alloys: A Review of Structure and Properties and Comparative Analysis with Other Available Techniques. J. Prosthodont..

[44] Al Jabbari Y.S., Koutsoukis T., Barmpagadaki X., Zinelis S., Al Jabbari Y.S., Koutsoukis T., Barmpagadaki X., Zinelis S. (2014). Metallurgical and interfacial characterization of PFM Co–Cr dental alloys fabricated via casting, milling or selective laser melting. Dent. Mater..

[45] Ghadhban A.H., Hasan I.H. (2022). Hardness and Surface Roughness of Cobalt-Chromium Alloy Produced by Selective Laser Melting and Casting Techniques (An in vitro study). J. Res. Med. Dent. Sci..

[46] Lapcevic A.R., Jevremovic D.P., Puskar T.M., Williams R.J., Eggbeer D. (2016). Comparative analysis of structure and hardness of cast and direct metal laser sintering produced Co-Cr alloys used for dental devices. Rapid Prototyp. J..

[47] Han X., Sawada T., Schille C., Schweizer E., Scheideler L., Geis-Gerstorfer J., Rupp F., Spintzyk S. (2018). Comparative analysis of mechanical properties and metal-ceramic bond strength of Co-Cr dental alloy fabricated by different manufacturing processes. Materials.

[48] Yu J.-M., Kang S.-Y., Lee J.-S., Jeong H.-S., Lee S.-Y. (2021). Mechanical Properties of Dental Alloys According to Manufacturing Process. Materials.

[49] Ko K.H., Kang H.G., Huh Y.H., Park C.J., Cho L.R. (2022). Effects of heat treatment on the microstructure, residual stress, and mechanical properties of Co-Cr alloy fabricated by selective laser melting. J. Mech. Behav. Biomed. Mater..

[50] Yamanaka K., Mori M., Chiba A. (2015). Assessment of precipitation behavior in dental castings of a Co-Cr-Mo alloy. J. Mech. Behav. Biomed. Mater..

[51] Maksimovic V.M., Stoiljkovic M.M., Čairovic A.D. (2016). Some consequences of repeated casting of Co-Cr dental alloy. J. Serbian Chem. Soc..

[52] Myszka D., Skrodzki M. (2016). Comparison of Dental Prostheses Cast and Sintered by SLM from Co-Cr-Mo-W Alloy. Arch. Foundry Eng..

[53] Fu W., Liu S., Jiao J., Xie Z., Huang X., Lu Y., Liu H., Hu S., Zuo E., Kou N. (2022). Wear Resistance and Biocompatibility of Co-Cr Dental Alloys Fabricated with CAST and SLM Techniques. Materials.

[54] Lee W.F., Wang J.C., Hsu C.Y., Peng P.W. (2022). Microstructure, mechanical properties, and retentive forces of cobalt-chromium removable partial denture frameworks fabricated by selective laser melting followed by heat treatment. J. Prosthet. Dent..

[55] Chimmat M., Srinivasan D. (2019). Understanding the Residual Stress in DMLS CoCrMo and SS316L using X-ray diffraction. Procedia Struct. Integr..

[56] Okazaki Y., Ishino A., Higuchi S. (2019). Chemical, physical, and mechanical properties and microstructures of laser-sintered Co-25Cr-5Mo-5W (SP2) and W-Free Co-28Cr-6Mo alloys for dental applications. Materials.

[57] Wei W., Zhou Y., Sun Q., Li N., Yan J., Li H., Liu W., Huang C. (2020). Microstructures and Mechanical Properties of Dental Co-Cr-Mo-W Alloys Fabricated by Selective Laser Melting at Different Subsequent Heat Treatment Temperatures. Metall. Mater. Trans. A.

[58] Zangeneh S., Erisir E., Abbasi M., Ramazani A. (2019). Evaluation of the aging effect on the microstructure of Co-28Cr-6Mo-0.3C alloy: Experimental characterization and computational thermodynamics. Metals.

[59] Roudnicka M., Bigas J., Molnarova O., Palousek D., Vojtech D. (2021). Different response of cast and 3D-printed Co-Cr-Mo alloy to heat treatment: A thorough microstructure characterization. Metals.

[60] Isik M., Niinomi M., Cho K., Nakai M., Liu H., Yilmazer H., Horita Z., Sato S., Narushima T. (2016). Microstructural evolution and mechanical properties of biomedical Co-Cr-Mo alloy subjected to high-pressure torsion. J. Mech. Behav. Biomed. Mater..

[61] Motra H.B., Hildebrand J., Dimmig-Osburg A. (2014). Assessment of strain measurement techniques to characterise mechanical properties of structural steel. Eng. Sci. Technol. Int. J..

[62] Hegele P., Von Kobylinski J., Hitzler L., Krempaszky C., Werner E. (2021). In-situ XRD study of phase transformation kinetics in a Co-Cr-W-alloy manufactured by laser powder-bed fusion. Crystals.

[63] von Kobylinski J., Hitzler L., Lawitzki R., Krempaszky C., Öchsner A., Werner E. (2020). Relationship between Phase Fractions and Mechanical Properties in Heat-Treated Laser Powder-Bed Fused Co-Based Dental Alloys. Isr. J. Chem..

[64] Sahami-Nejad M., Lashgari H.R., Zangeneh S., Kong C. (2019). Determination of residual stress on TIG-treated surface via nanoindentation technique in Co-Cr-Mo-C alloy. Surf. Coat. Technol..

[65] Lambrou I., Kaldellis A., Stergiou V., Tsakiridis P.E. (2022). Characterisation of heat-treated cobalt—Chromium alloy fabricated by selective laser melting. Mater. Sci. Technol..

[66] Zhou Y., Sun Q., Dong X., Li N., Shen Z.J., Zhong Y., Eriksson M., Yan J., Xu S., Xin C. (2020). Microstructure evolution and mechanical properties improvement of selective laser melted Co-Cr biomedical alloys during subsequent heat treatments. J. Alloys Compd..

[67] Vander Sande J.B., Coke J.R., Wulff J. (1976). A transmission electron microscopy study of the mechanisms of strengthening in heat-treated Co-Cr-Mo-C alloys. Metall. Trans. A.

